# The Effect of Letrozole Combined with Dydrogesterone for Endometriosis in China: A Meta-Analysis

**DOI:** 10.1155/2021/9946060

**Published:** 2021-12-02

**Authors:** Shumei Sun, Hao Zhang, Peicheng Zhong, Zhihong Xu

**Affiliations:** ^1^School of Medical and Life Sciences, Chengdu University of Traditional Chinese Medicine, Chengdu, 610000 Sichuan, China; ^2^People's Hospital of Deyang City, Deyang, 618000 Sichuan, China; ^3^Huizhou Municipal Central Hospital, Huizhou, 516000 Guangdong, China

## Abstract

**Purpose:**

To discuss the effects of dydrogesterone combined with letrozole on the effectiveness, sex hormone levels, and serological indicators in patients with endometriosis. This study is registered with PROSPERO (CRD42020213172).

**Methods:**

We searched relevant randomized controlled trials (RCTs) through PubMed, Cochrane Library, China National Knowledge Infrastructure (CNKI), Wanfang, and VIP Database. The standardized mean differences (SMDs), the mean differences (MDs), or odds ratios (ORs) with their 95% confidence intervals (95% CIs) were computed to be outcome indicators, including total effectiveness, Vascular Endothelial Growth Factor (VEGF) level, Carbohydrate Antigen 125 (CA125) level, Follicle-Stimulating Hormone (FSH) level, Luteinizing Hormone (LH) level, estrogen (E2) level, progesterone (P) level, interleukin-6 (IL-6) level, and tumor necrosis factor-a (TNF-a) level.

**Results:**

A total of 19 RCTs involving 1,591 patients were included in this study. Our results showed that letrozole combined with dydrogesterone can significantly reduce the levels of VEGF (SMD -2.23, 95% CI -2.39 to -2.07; *p* < 0.00001), CA125 (MD -10.53, 95% CI -11.19 to -9.88; *p* < 0.00001), E2 (SMD -1.64, 95% CI -1.81 to -1.47; *p* < 0.00001), P (MD -5.11, 95% CI -6.26 to -3.96; *p* < 0.00001), IL-6 (MD -4.41, 95% CI -5.16 to -3.67; *p* < 0.00001), and TNF-a (MD -5.67, 95% CI -6.34 to -5.00; *p* < 0.00001) in patients with endometriosis compared with the control group. In addition, the results indicated that total effectiveness was significantly higher in the experiment group (OR 6.21, 95% CI 4.17 to 9.24; *p* < 0.00001) compared to the control. However, there was no significant difference between FSH and LH levels in both groups (*p* > 0.05).

**Conclusion:**

This combination therapy can effectively decrease the levels of VEGF, CA125, E2, P, IL-6, and TNF-a and increase the total effectiveness when comparing with the control group.

## 1. Introduction

Endometriosis, a debilitating disease characterized by chronic inflammation, is defined as the presence of functional endometrial glands and stroma outside the uterine cavity, and the most common sites are the ovaries, the uterosacral ligaments, and the posterior cul-de-sac [1], while the rare parts are the diaphragm, pleura, and pericardium [2, 3]. According to statistics, the incidence of endometriosis in women of childbearing age is as high as 10% and as high as 35–50% of women with pelvic pain or infertility [4]. It has been reported in the literature that patients with endometriosis are more anxious than those without endometriosis, and the mechanism is that the disease affects the passage of cell membrane magnesium ion [5]. Endometriosis is an estrogen-dependent disorder [6] that can result in a variety of symptoms, including dysmenorrhea or menorrhagia, pain during or after sexual intercourse, abdominal pain, or infertility. The pathogenesis of endometriosis has not yet been clarified, and there is no effective medicine to treat it. Therefore, the focus of treatment is relieving symptoms rather than curing [7]. It contains drug treatment and surgical treatment. Clinically, mifepristone is mainly used for the treatment of endometriosis, but there is insufficient evidence to determine the safety and effectiveness of other progesterone receptor modulators [8]. However, there are many literatures that reported that aromatase inhibitors (such as letrozole) are effective in relieving endometriosis associated with chronic pelvic pain in both reproductive-aged and postmenopausal women [9–11]. In addition, dydrogesterone has been effective in the symptomatic management of pain and other symptoms caused by endometriosis [12]. Hence, we performed this meta-analysis to further elucidate the role of letrozole combined with dydrogesterone in endometriosis, providing clues for developing novel therapies for endometriosis.

## 2. Methods

This meta-analysis has been registered on the PROSPERO website (https://www.crd.york.ac.uk/prospero/), of which the registration number is CRD42020213172. Details can be found on the website.

### 2.1. Search Strategy

We searched the PubMed, Cochrane Library, China National Knowledge Infrastructure (CNKI), and Wanfang databases as well as VIP Database from their inception until 2020 for correlative articles by various combinations of terms including aromatase inhibitors, letrozole, dydrogesterone, progestogen, and endometriosis. It was eligible for inclusion in our research if the article was written in Chinese or English.

### 2.2. Inclusion and Exclusion Criteria

Inclusion criteria were as follows: (1) randomized controlled trials (RCTs) of letrozole combined with dydrogesterone for endometriosis; (2) the subjects of studies were women with confirmed endometriosis; (3) studies contained the experimental group and the control group; (4) the control group was treated with letrozole alone while the experimental group was treated with dydrogesterone in combination with letrozole. Exclusion criteria were as follows: (1) articles of nonrandomized controlled trials; (2) no control group; (3) animal and cell studies; (4) repeated publications; (5) the content of the study was not consistent with the theme.

### 2.3. Data Extraction

Data extraction was performed by two reviewers. The following data were extracted: first author, year of publication, study design, the number of people in the control group and the experimental group, baseline characteristics and interventions of study subjects, outcome indicators (such as total effectiveness, Vascular Endothelial Growth Factor (VEGF) level, Carbohydrate Antigen 125 (CA125) level, Follicle-Stimulating Hormone (FSH) level, Luteinizing Hormone (LH) level, estrogen (E2) level, progesterone (P) level, interleukin-6 (IL-6) level, and tumor necrosis factor-a (TNF-a) level), and outcome measures of concern.

### 2.4. Quality Assessment

The literatures included in this study were evaluated by the Cochrane quality assessment tool [13], and its risk of bias was assessed, containing random sequence generation (selection bias), allocation concealment (selection bias), blinding of participants and personnel (performance bias), blinding of outcome assessment (detection bias), incomplete outcome data (attrition bias), selective reporting (reporting bias), and other bias. In addition, risk of bias graph and risk of bias summary were mapped for inclusion in the study.

### 2.5. Data Synthesis and Analysis

For outcome indicators such as VEGF level, FSH level, LH level, and E2 level, we used the standardized mean differences (SMDs) and 95% confidence intervals (95% CIs), while we used the mean difference (MDs) and 95% confidence intervals (95% CIs) for outcome indicators, including CA125 level, P, IL-6 level, and TNF-a level. Odds ratios (ORs) with 95% CIs were used to calculate total effectiveness after combining letrozole and dydrogesterone to treat endometriosis. We evaluated statistical heterogeneity by using the Cochran chi-square (*X*^2^) and the *I*^2^ statistic. The random-effects model was adopted when heterogeneity was high (*I*^2^ > 50%). A *p* value less than 0.1 was considered as significant heterogeneity for the *X*^2^ test. We performed sensitivity analysis to explore the source of heterogeneity and to evaluate the impact of a single study on the results. All statistical tests were bilateral tests, and statistical significance was defined as a *p* value less than 0.05. We used Review Manager 5.1 for statistical analyses.

This paper is a systematic analysis based on research data, so it does not need ethical approval.

## 3. Results

### 3.1. Study Selection and Study Characteristics

**Figure 1:**
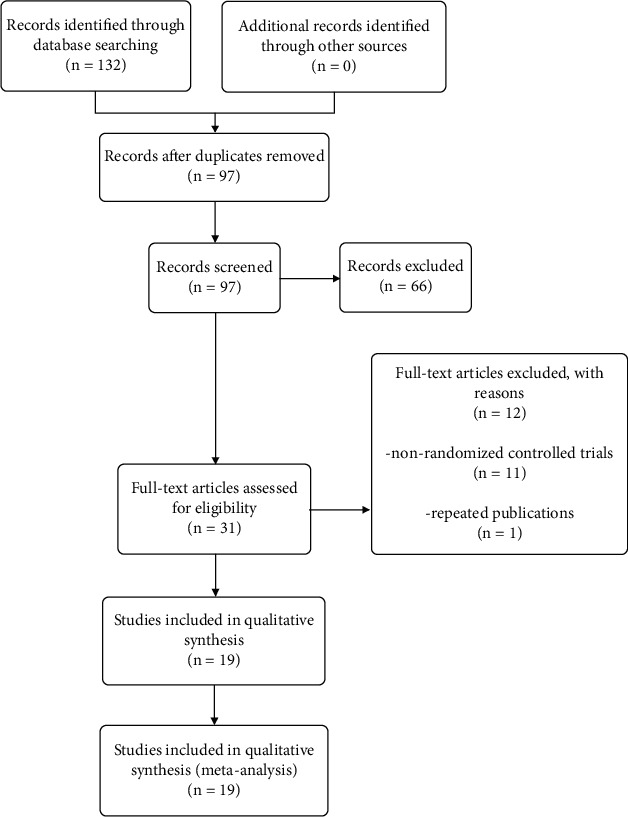
Flow diagram of the search process.

As shown in Figure 1, a total of 132 records were retrieved. 35 studies were excluded because they were duplicates. 66 articles were excluded by reading titles and abstracts. 12 were excluded due to the nonrandomized controlled trials (*n* = 11) and the repeated publications (*n* = 1). Therefore, 19 articles, involving 1591 women, were analyzed. All of the patients were Chinese that included 796 patients in the experimental group and 795 patients in the control group. The details of those studies are given in Table 1.

## 4. Meta-Analysis Results

### 4.1. Meta-Analysis of Total Effectiveness

**Figure 2:**
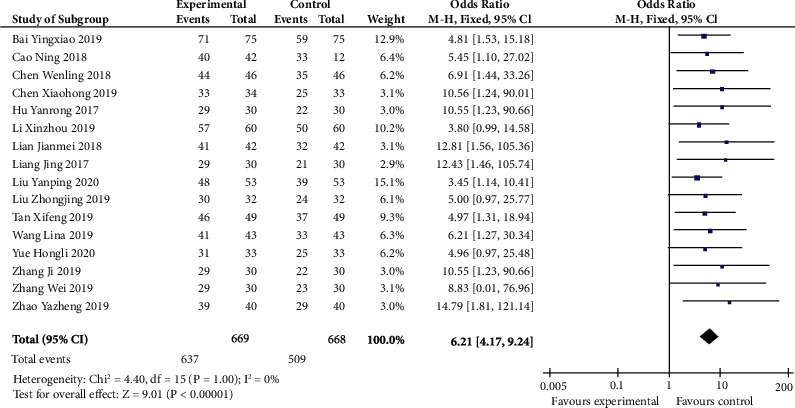
Total effectiveness (forest plot meta-analysis).

Sixteen studies calculated total effectiveness of letrozole combined with dydrogesterone in the treatment of endometriosis. Compared with the control group, the result indicated that total effectiveness was significantly higher in the experiment group (OR 6.21, 95% CI 4.17 to 9.24; *p* < 0.00001) (Figure 2). There was no heterogeneity among studies (*p* 1.00, *I*^2^ 0%).

### 4.2. Meta-Analysis of VEGF Level

**Figure 3:**
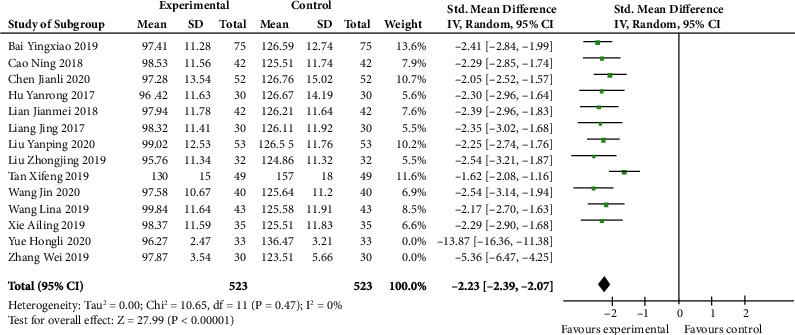
VEGF level after sensitivity analysis (forest plot meta-analysis).

Twelve articles were analyzed in this part. VEGF level was significantly lower in the experiment group than in the control group (SMD -2.23, 95% CI -2.39 to -2.07; *p* < 0.00001) (Figure 3). In addition to these literatures, there were two studies conducted by Yue Hongli or Zhang Wei that also contained this index. However, heterogeneity was high between studies (*I*^2^ 89%) if these two studies were included. Therefore, according to the sensitivity analysis, these two researches were removed and the heterogeneity reduced from 89% to 0% (*p* 0.47, *I*^2^ 0%). These two studies may be potential sources of heterogeneity.

### 4.3. Meta-Analysis of CA125 Level

**Figure 4:**
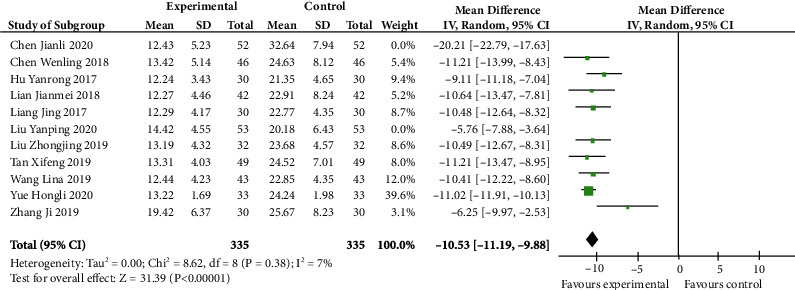
CA125 level after sensitivity analysis (forest plot meta-analysis).

As shown in Figure 4, there were nine articles investigating the CA125 results of using letrozole combined with dydrogesterone to cure endometriosis. The CA125 level was markedly less in the experiment group than in the control group (MD -10.53, 95% CI -11.19 to -9.88; *p* < 0.00001). Similarly, in addition to these literatures, there were two studies conducted by Chen Jianli or Liu Yanping that also contained this index. However, the heterogeneity was still significant (*I*^2^ 88%) if these two studies were included. Thus, according to the sensitivity analysis, the heterogeneity decreased from 88% to 7% (*p* 0.38, *I*^2^ 7%) after removing the researches. Those two studies may be potential sources of heterogeneity.

### 4.4. Meta-Analysis of FSH Level

**Figure 5:**
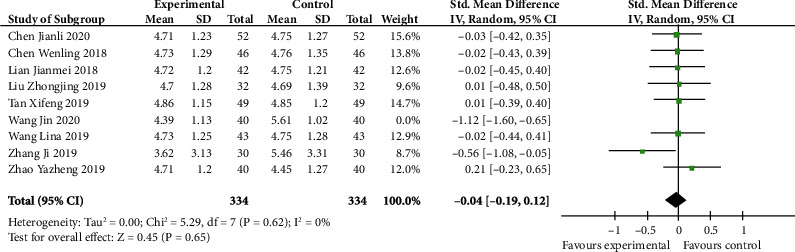
FSH level after sensitivity analysis (forest plot meta-analysis).

As we can see in Figure 5, no differences were found in FSH level in both groups (SMD -0.04, 95% CI -0.19 to 0.12; *p* 0.65). The heterogeneity was huge (*I*^2^ 66%). Nonetheless, when we performed the sensitivity analysis by removing the research of Wang Jin, the heterogeneity was reduced (*p* 0.62, *I*^2^ 0%). This was likely to be a source of heterogeneity.

### 4.5. Meta-Analysis of LH Level

**Figure 6:**
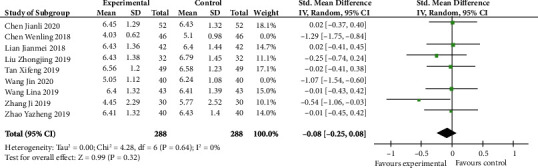
LH level after sensitivity analysis (forest plot meta-analysis).

LH level, between letrozole combined with dydrogesterone and letrozole alone in the treatment of endometriosis, is demonstrated in Figure 6 (SMD -0.08, 95% CI -0.25 to 0.08; *p* 0.32). The heterogeneity stayed significant (*I*^2^ 80%) if we did not do the sensitivity analysis. However, according to the sensitivity analysis, after removing researches conducted by Chen Wenling and Wang Jin, the result of the meta-analysis changed and showed that there was no significant difference in both groups (*p* 0.32) and the heterogeneity disappeared (*p* 0.64, *I*^2^ 0%). Those two studies possibly are potential sources of heterogeneity.

### 4.6. Meta-Analysis of E2 Level

**Figure 7:**
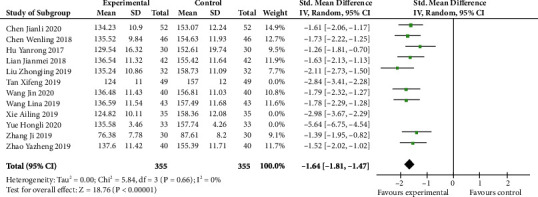
E2 level after sensitivity analysis (forest plot meta-analysis).

Figure 7 shows that E2 was markedly higher in the control group than in the experiment group (SMD -1.64, 95% CI -1.81 to -1.47; *p* < 0.00001). We found that the heterogeneity was huge (*I*^2^ 86%). Therefore, according to the sensitivity analysis, because we tried to remove some studies (such as Tan Xifeng, Xie Ailing, and Yue Hongli), the heterogeneity disappeared (*p* 0.66, *I*^2^ 0%). Hence, we thought those three articles might be potential sources of heterogeneity.

### 4.7. Meta-Analysis of P Level

**Figure 8:**
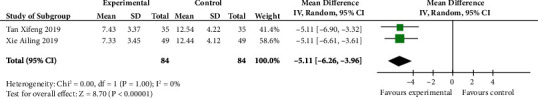
P level (forest plot meta-analysis).

P level after using letrozole combined with dydrogesterone to influence endometriosis is given in Figure 8. Not only the experiment group was discovered less than the control group in P level (MD -5.11, 95% CI -6.26 to -3.96; *p* < 0.00001) but also there was no heterogeneity between studies (*p* 1.00, *I*^2^ 0%).

### 4.8. Meta-Analysis of IL-6 Level

**Figure 9:**
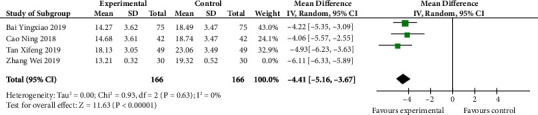
IL-6 level after sensitivity analysis (forest plot meta-analysis).

In the following picture (Figure 9), we can know that the IL-6 level was striking smaller in experiment than in control (MD -4.41, 95% CI -5.16 to -3.67; *p* < 0.00001). The heterogeneity was significant (*I*^2^ 84%), so we eliminated the study of Zhang Wei and then the heterogeneity vanished (*p* 0.63, *I*^2^ 0%). Therefore, we had a reason to believe that this was a major source of heterogeneity.

### 4.9. Meta-Analysis of TNF-a Level

**Figure 10:**
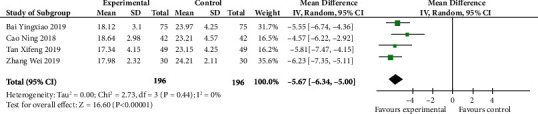
TNF-a level (forest plot meta-analysis).

According to Figure 10, we learned that the level of TNF-*α* in the experimental group was significantly lower than that in the control group (MD -5.67, 95% CI -6.34 to -5.00; *p* < 0.00001). Moreover, no heterogeneity was understood between studies (*p* 0.44, *I*^2^ 0%).

### 4.10. Risk of Bias

**Figure 11:**
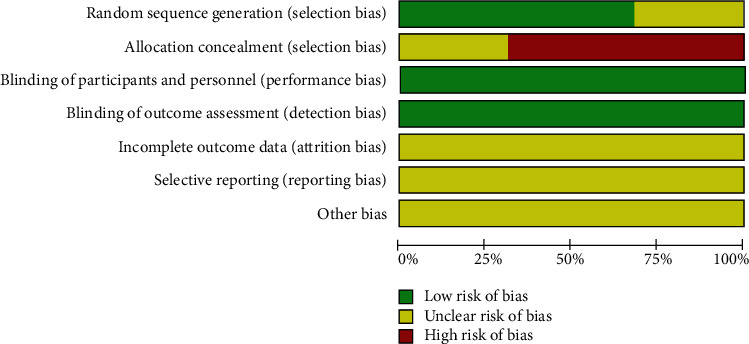
Risk of bias graph.

**Figure 12:**
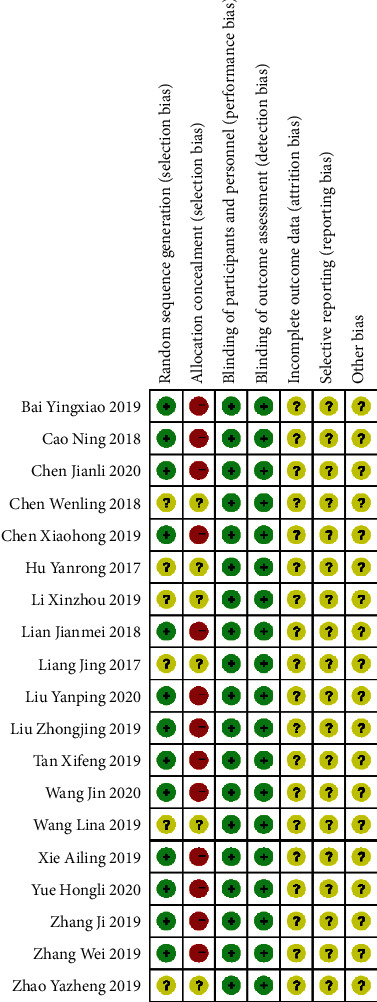
Risk of bias summary.

All studies were assessed for risk of bias using the Cochrane quality assessment tool [13]. The results were presented through risk of bias graph (Figure 11) and risk of bias summary (Figure 12).

Regarding random sequence generation, there were thirteen studies with sufficient random sequence generation, as described in the relevant articles [14–17, 19, 21, 23–26, 29, 30, 32], and therefore were judged to have low risk of bias. On the contrary, the other six studies [18, 20, 22, 27, 28, 31] did not explain how the sequence was generated and were therefore assessed as an uncertain risk of bias.

With regard to allocation concealment, twelve studies showed that allocation process was made by using a random number table so that they were considered to be at high risk of bias [14–17, 19, 21, 23–26, 29, 32]. By contrast, the rest of the studies [18, 20, 22, 27, 28, 30, 31] did not describe how the allocation process was carried out. Hence, they were thought of as having uncertain risk of bias.

Concerning blinding of participants and outcome assessment, because lack of blinding did not seem to compromise the outcomes, all studies with no attempted masking for either participants or outcome assessment were deemed as an uncertain risk of bias.

Considering incomplete outcome data, all studies did not completely illustrate data loss and were considered to be at uncertain risk of bias. In regard to selective reporting, all articles also did not describe this information in details so they were an uncertain risk of bias.

None of the studies had enough information to assess whether they were at risk of other significant biases.

## 5. Discussion

Endometriosis is a disease that occurs at birth with a high incidence of reproductive age. Studies have shown that endometriosis is a “heritable, hormone-dependent gynecological disorder” disease [33, 34]. Although endometriosis is morphologically benign, clinical ethology is similar to malignant tumors considering its implantation, invasion, and distant metastasis. Therefore, the treatment of endometriosis remains a challenge. It has been suggested that the increase of aromatase activity in endometriosis tissue results in local estrogen production and the growth of endometriosis, which is associated with disease symptoms such as pelvic pain [35]. So we assumed that aromatase inhibitors can inhibit the synthesis of estrogen in the ovary.

Letrozole, a third-generation aromatase inhibitor which is the most potent, highly selective, and reversible, is a drug that inhibits the aromatase enzyme by competitively binding to the cytochrome P 450 subunit of the enzyme, giving rise to suppression of estrogen biosynthesis in all tissues [36]. Dydrogesterone is an oral progesterone that allows the endometrium to enter a fully secretory phase in order to prevent the risk of endometrial hyperplasia and cancer caused by estrogen. It has been reported that dydrogesterone can relieve the symptoms of endometriosis, alleviate the lesions, and improve the pregnancy rate of infertile patients [37, 38].

In this study, the total effectiveness of the experiment group was higher than that of the control group while the incidence of total adverse reactions was significantly lower in the experiment group than in the control group. It was probable because the basis of dydrogesterone combined with letrozole can directly affect the aromatase of the focus to significantly inhibit its activity and then affect the expression of estrogen at a lower level so as to achieve its effective treatment. These results suggested that dydrogesterone combined with letrozole was safe and effective in improving the clinical symptoms of endometriosis.

According to the report, angiogenesis plays a crucial role in the formation of endometriosis lesions and the growth of ectopic sites [39]. Accordingly, endometriosis may be treated with less VEGF production. In addition, CA125 is a glycoprotein that can be bound by monoclonal antibody OC125, detecting from epithelial ovarian cancer antigen. It is widely accepted that serum CA125 concentrations were elevated in women with endometriosis [40] and CA125 can be expressed in endometriotic tissue [41]. Moreover, Wang et al. found that the IL-6 expression exhibited an increase in endometriosis [42]. Tumor necrosis factor, a factor in the serum, can kill certain tumor cells or cause blood necrosis in tumor tissues in the body. Some articles have demonstrated that TNF-*α* has an important role in the development of endometriosis [43].

This study showed that letrozole combined with dydrogesterone can significantly reduce the levels of VEGF, CA125, E2, P, IL-6, and TNF-a in patients with endometriosis compared with the control group. The results suggested that the treatment of endometriosis with dydrogesterone combined with letrozole can improve the serum sex hormone levels and serological indicators and further lead to shorten the course of endometriosis patients and determine the therapeutic effect. However, there was no significant difference in FSH and LH level between the drug combination and the single drug. In addition to the above indicators, it was reported that compared with normal-weight women, the incidence of endometriosis in obese women has increased significantly, but there is no correlation between the relevant quantitative indicator (body mass index, BMI) and endometriosis. Affected by this limitation, this study did not use the BMI to evaluate the combined effect of letrozole combined with dydrogesterone [44, 45].

Despite aromatase inhibitors having a good effect on the treatment of endometriosis, some scholars do not believe that aromatase is as important in the pathology of endometriosis as previously assumed, at least not in the endometriosis lesions themselves [46]. What is more, the side effects of aromatase inhibitors, such as osteoporosis, are not negligible. Therefore, aromatase inhibitors are always used in combination with other drugs (mainly progestins, oral contraceptives, and gonadotropin-releasing hormone (GnRH) agonists), which makes the combination of drugs in this paper possible.

## 6. Limitations

There are several limitations that should be mentioned to interpret the results in this study. Firstly, there was great heterogeneity between studies so we performed sensitivity analysis to explore the sources of heterogeneity. These may be due to the sample size and the measuring method. Secondly, in the study, English literatures that met the inclusion criteria were not retrieved, which may have affected the extrapolation of the results. Last but not the least, although all the included studies explained randomness, some did not point out specific implementation methods, which may exist as performance bias.

## 7. Conclusions

Our study suggested that letrozole combined with dydrogesterone was more effective than letrozole alone for the treatment of endometriosis. It was mainly manifested in the decrease of VEGF, CA125, E2, P, IL-6, and TNF-a as well as in the increase of the total effectiveness. Therefore, this information may help us to learn that this combination therapy may be considered for the management of endometriosis in the future. However, limited by the quantity and quality of the included studies, the above conclusions need to be verified by more high-quality studies.

## Figures and Tables

**Table 1:** Details of included studies.

Source	Study design	Number (T/C)	Age (T/C)	Interventions(T/C)	Intervention time	Outcome indicators
Liu Yanping 2020 [14]	RCT	53/53	33.08 ± 5.69/33.05 ± 5.67	Letrozole combined with dydrogesterone/letrozole	6 months	①②③
Wang Jin 2020 [15]	RCT	40/40	35.04 ± 3.61/35.14 ± 3.54	Letrozole combined with dydrogesterone/letrozole	6 months	②④⑤⑥
Zhang Ji 2019 [16]	RCT	30/30	35.72 ± 3.91/34.56 ± 3.84	Letrozole combined with dydrogesterone/letrozole	6 months	①③④⑤⑥
Xie Ailing 2019 [17]	RCT	35/35	30.28 ± 3.41/30.53 ± 3.25	Letrozole combined with dydrogesterone/letrozole	6 months	②⑥⑦
Li Xinzhou 2019 [18]	RCT	60/60	29.56 ± 4.78/28.98 ± 4.26	Letrozole combined with dydrogesterone/letrozole	6 months	①
Liu Zhongjing 2019 [19]	RCT	32/32	39.79 ± 6.32/38.74 ± 5.21	Letrozole combined with dydrogesterone/letrozole	6 months	①②③④⑤⑥
Zhao Yazheng 2019 [20]	RCT	40/40	33.01 ± 7.14/33.10 ± 7.15	Letrozole combined with dydrogesterone/letrozole	6 months	①④⑤⑥
Zhang Wei 2019 [21]	RCT	30/30	35.26 ± 3.12/35.54 ± 3.54	Letrozole combined with dydrogesterone/letrozole	6 months	①②⑧⑨
Wang Lina 2019 [22]	RCT	43/43	-	Letrozole combined with dydrogesterone/letrozole	6 months	①②③④⑤⑥
Bai Yingxiao 2019 [23]	RCT	75/75	31.47 ± 4.72/31.26 ± 4.95	Letrozole combined with dydrogesterone/letrozole	6 months	①②⑧⑨
Tan Xifeng 2019 [24]	RCT	49/49	33.0 ± 2.7/33.3 ± 2.7	Letrozole combined with dydrogesterone/letrozole	6 months	①②③④⑤⑥⑦⑧⑨
Cao Ning 2018 [25]	RCT	42/42	34.24 ± 1.74/34.59 ± 1.56	Letrozole combined with dydrogesterone/letrozole	6 months	①②⑧⑨
Lian Jianmei 2018 [26]	RCT	42/42	32.73 ± 6.12/33.12 ± 5.74	Letrozole combined with dydrogesterone/letrozole	6 months	①②③④⑤⑥
Chen Wenling 2018 [27]	RCT	46/46	32.58 ± 4.17/33.02 ± 4.37	Letrozole combined with dydrogesterone/letrozole	6 months	①③④⑤⑥
Hu Yanrong 2017 [28]	RCT	30/30	32.84 ± 2.57/33.16 ± 2.61	Letrozole combined with dydrogesterone/letrozole	6 months	①②③⑥
Chen Xiaohong 2019 [29]	RCT	34/33	29.70 ± 2.15/29.43 ± 2.18	Letrozole combined with dydrogesterone/letrozole	6 months	①
Yue Hongli 2020 [30]	RCT	33/33	32.51 ± 4.26/33.46 ± 2.51	Letrozole combined with dydrogesterone/letrozole	6 months	①②③⑥
Liang Jing 2017 [31]	RCT	30/30	33.01 ± 6.42/32.14 ± 6.51	Letrozole combined with dydrogesterone/letrozole	6 months	①②③
Chen Jianli 2020 [32]	RCT	52/52	32.84 ± 2.71/32.93 ± 2.70	Letrozole combined with dydrogesterone/letrozole	6 months	②③④⑤⑥

T: the experiment group; C: the control group; -: not mentioned; RCT: randomized controlled trial; ①: total effectiveness; ②: VEGF level; ③: CA125 level; ④: FSH level; ⑤: LH level; ⑥: E2 level; ⑦: P level; ⑧: IL-6 level; ⑨: TNF-a level; VEGF: Vascular Endothelial Growth Factor; CA125: Carbohydrate Antigen 125; FSH: Follicle-Stimulating Hormone; LH: Luteinizing Hormone; E2: estrogen; P: progesterone; IL-6: interleukin-6; TNF-a: tumor necrosis factor-a.

## Data Availability

All the data for this article are contained in the manuscript.

## Abbreviations

RCTs: Randomized controlled trials
SMDs: Standardized mean differences
MDs: Mean differences
ORs: Odds ratios
95% CIs: 95% confidence intervals
VEGF: Vascular Endothelial Growth Factor
CA125: Carbohydrate Antigen 125
FSH: Follicle-Stimulating Hormone
LH: Luteinizing Hormone
E2: Estrogen
P: Progesterone
IL-6: Interleukin-6
TNF-a: Tumor necrosis factor-a.
